# Barriers and facilitators to shared decision making in child and youth mental health: clinician perspectives using the Theoretical Domains Framework

**DOI:** 10.1007/s00787-018-1230-0

**Published:** 2018-09-18

**Authors:** D. Hayes, J. Edbrooke-Childs, R. Town, M. Wolpert, N. Midgley

**Affiliations:** 1 Evidence Based Practice Unit, University College London and Anna Freud Centre, 21 Maresfield Gardens, London, NW3 5SD UK; 2 Anna Freud National Centre for Children and Families, 21 Maresfield Gardens, London, NW3 5SD UK

**Keywords:** Shared decision making, Patient preference, Clinician perspectives, Theoretical Domains Framework (TDF), Qualitative research

## Abstract

Shared decision making (SDM) is increasingly being suggested as an integral part of mental health provision. Yet, there is little research on what clinicians believe the barriers and facilitators around practice to be. At the same time, there is also increasing recognition of a theory–practice gap within the field, with calls for more pragmatic uses of theory to inform and improve clinical practice. Using the Theoretical Domains Framework (TDF), a comprehensive, theoretical-led framework, underpinned by 33 behaviour change theories and 128 constructs, clinician perceived barriers and facilitators to SDM are investigated. The sample comprised of 15 clinicians across two sites in England, who took part in qualitative semi-structured interviews and focus groups. Transcripts were analysed using a deductive thematic analysis, and themes were coded under each theoretical domain. Overall, 21 barriers and facilitators for SDM in child and youth mental health were identified across ten domains of the TDF. Under capability, barriers and facilitators were found for knowledge, skills, memory/attention/decision making processes, and behavioural regulation. For opportunity, barriers and facilitators were found for social influences, as well as environmental context and resources. Finally, for motivation, domains covered included: beliefs about consequences, beliefs about capabilities, emotions, and professional role and identity. Findings suggest that a range of barriers and facilitators affect clinicians’ abilities to engage in SDM with young people and parents. Interventions which target different domains related to capability, opportunity and motivation should be developed to better facilitate young people and their families in care and treatment decisions.

**Electronic supplementary material:**

The online version of this article (10.1007/s00787-018-1230-0) contains supplementary material, which is available to authorized users.

## Introduction

Shared decision making (SDM) is defined as the process in which clinicians and patients work together to come up with decisions about their care or treatment [1, 2]. Conversations around SDM may focus on, but not be limited to: tests for screening, undergoing procedures, participating in self-help or psychological interventions, whether to take medication, and whether to make changes to the patient’s lifestyle [2]. Importantly, SDM highlights that both patients and clinicians have something to contribute to the conversation; the clinician having medical knowledge and clinical experience, and the patient having knowledge of themselves and what would fit with their lifestyles [1].

Internationally, there is increasing interest in SDM [3]. For young people, the right to be involved in healthcare decisions is underpinned by Articles 12 and 13 of the United Nations Convention of the Rights of the Child, which outlines that young people’s views should be taken in line with their age and maturity. This has translated through to many Western Countries, with the USA, Netherlands, Australia and UK advocating and promoting ways of involving young people and families in care and treatment decisions [4–7].

Research into interventions to support SDM in child and youth mental health is an expanding area [6]. A recent scoping review identified 23 different approaches split into six overarching categories: decision aids, psychoeducational information, mobilizing patients to engage, therapeutic techniques, action planning and goal setting, and discussion prompts [6]. However, many interventions in this field lack an explicit theoretical basis [8]. This is indicative of the wider theory–practice gap in SDM, whereby it is posited that one single theory, often focusing on cognitive elements of decision making, are unlikely to be sufficient, as they neglect other aspects, such as the environment and decision making tools [9]. The Theoretical Domains Framework [10], which is an amalgamation of 33 behaviour change theories, exploring barriers and facilitators across fourteen unique domains, has been proposed as a solution to the theory–practice gap [8] as it can help identify theoretically driven targets for interventions.

The scoping review also identified that most approaches to SDM tend to focus on externalising difficulties [6]. However, research suggests that increasing severity of both internalising and externalising difficulties of young people has been found to predict SDM [8, 11–13]. There is increasing evidence of rising rates of internalising difficulties in youth, particularly in girls, with reporting raised anxiety and depression [14, 15]. Anxiety and depression are the most frequently difficulties amongst youth seeking help from specialist mental health services [16]. Thus, finding ways which young people with internalising difficulties can be involved in decision making is important. Particularly, as it has been posited that SDM may help develop safer care in mental health with young people [17].

There is limited research into what clinicians believe the barriers and facilitators to be when it comes to SDM with young people with mental health difficulties. Reviews that exist tend to focus on related concepts such as person-centred care [18] or have been extrapolated from experience of SDM, rather than setting out to explore barriers and facilitators [19–23]. In the research that does exist, factors that can help or hinder SDM have focused on whether young people have the capacity to be involved [19, 21, 23], whether services and clinicians can be flexible when it comes to treatment [19, 20, 22, 23], and if clinicians are able to listen, respect, and validate the young person [20, 22]. Research also suggests that parents can be a barrier or facilitator depending on whether they are supportive or not of the young person’s decision [20, 23].

Only one study has specifically set out to explore clinician perceived barriers and facilitators to decision making for young people [24]. Findings for barriers and facilitators included: the availability of treatment options, what information resources were available and whether these were age appropriate, whether there was an evidence base for the treatment, if professionals involved agreed with the treatment plan amongst themselves, whether the clinicians were willing to talk about side effects, the team culture around decision making, and factors relating to information sharing and confidentiality. These were conducted within an Australian healthcare system, which has some substantial differences compared with the UK.

Much of the research to date has had no explicit aim of exploring barriers and facilitators to SDM and has been conducted mostly in relation to children with externalising difficulties. There is need for a study that explores the views of clinicians with regard to the barriers and facilitators to SDM, with a particular focus on work with young people with internalising difficulties.

### Aims of the present study

Based on the above, this study aimed to explore clinicians’ views of the barriers and facilitators around SDM with young people who have internalising disorders along with their parents, within two services in England.

## Methods

### Participants

Two English National Health Service (NHS) Trusts took part in this qualitative study. One NHS Trust (Site A) consisted of a single child and adolescent mental health service (CAMHS) based in the east of England. The second NHS Trust consisted of four CAMHS clinics based in the Midlands. Both consisted of both targeted and specialist services for young people (Tiers 2 and 3).

Fifteen clinicians from the two sites participated in this study. Seven clinicians were recruited from Site A and eight from Site B. The majority of the clinicians were recruited through convenience sampling (*n* = 11), whilst additional clinicians were recruited through snowball sampling (*n* = 4). The clinicians were aged between 27 and 55 years old *(M *= 43.11, SD = 9.65). Two identified as male (13%) and the remainder identified as female (87%). With regard to ethnic background, nine clinicians identified as White British (60%), four as Asian British (27%), one as Asian Indian (7%) and one as Black British (7%). In terms of professional groupings, there were five clinical psychologists (33%), three trainee psychologists (20%), two psychiatrists (13%), four mental health nurses (27%) and one CBT therapist (7%). The clinicians had been working in their professions between 3 months and 11 years. At the time of their interviews, all the clinicians worked in mental health services intended for young people aged 16 years or below. The clinician interviews lasted between 24 and 62 min (*M *= 38.24, SD = 8.89).

### Procedure

This research was undertaken by a male PhD candidate (DH) as part of his doctoral thesis and follows the COREQ reporting guidelines for qualitative studies [25]. The researcher was trained in qualitative methods and had experience conducting and analysing interviews and focus groups. The creation of the interview schedule was developed in accordance with the TDF [10]. Interview schedules were mock-tested with one clinician, one trainee clinician, and a doctoral researcher to ensure comprehension and clarity. No changes were made as a result of this testing. The TDF was chosen as it explores the barriers and facilitators to behavioural phenomena, whilst being underpinned by theoretical constructs. This may be one solution to the theory–practice gap outlined [9] which observes that theories fail to consider the multiple aspects of SDM.

The project was presented at team meetings and clinicians who expressed an interest in the project were provided with further information. Follow-up phone calls were arranged with interested participants to establish a rapport with them. Interviews were arranged at times convenient for the clinicians, which meant that they could take place either over the phone or in person. This resulted in five clinicians participating in face-to-face interviews at the service and five clinicians participating in telephone interviews. The remaining five clinicians participated in a focus group at their service due to logistical and time constraints. All the clinicians who participated in the focus group were from the same service (Site B), and content did not differ from other interviews conducted in the same service. Other than the focus group, all interviews were conducted with only the researcher and clinician present. The reasons why some clinicians did not want to take part were not explored.

The lead researcher (DH) undertook all data collection using an audio recorder. Prior to the interviews, the researcher outlined that the research was part of their doctoral thesis and that they were interested in clinicians’ positive and negative experiences of SDM. The clinicians were then asked questions corresponding to the TDF about what they believed to be the barriers and facilitators to SDM with young people with internalising difficulties and their parents. Prompts were provided for clinicians if needed (see interview schedule) and some reflections and field notes were taken. The discussions were transcribed verbatim. No follow-up interviews were conducted.

### Data analysis

The transcribed interviews were analysed using a thematic analysis [26] in NVivo [27]. This flexible method is used to identify patterns of meaning within data among participants and is not allied to any particular framework. As such, it may be used within any theoretical framework, as well as in the absence of one [26]. Braun and Clark [26] outline six steps that are undertaken as part of a thematic analysis. These consist of familiarising oneself with the data, the generation of codes, searching for themes, the reviewing of themes, defining and naming themes, and producing a report. A deductive approach was applied in the form of existing constructs (e.g., capability) and theoretical domains (e.g., skills). However, there was scope to define subthemes which arose within each domain.

Once the lead researcher (DH) developed a coding key in line with the TDF based on all transcribed interviews, a second researcher (RT) independently applied codes to four transcripts (27%) chosen at random. A good level of agreement (Kappa = 0.81) was obtained using this method. The themes from these data, rather than individual transcripts, were presented at clinician team meetings for further comment. No changes were made as a result of this.

### Ethical considerations

Ethical consideration was sought and obtained from the London Hampstead NRES Committee (REC ref: 15/LO/0997).

## Results

Overall, 21 subthemes across ten domains of the TDF [10] were identified as factors that were barriers or facilitators to SDM. These spanned all three areas related to capability, opportunity and motivation [28]. The results are highlighted in Table 1.

**Table 1:** Clinician barriers and facilitators to SDM using the TDF [10]

COM-B	TDF	Barriers and facilitators
Capability	Knowledge	An awareness of the philosophy of SDM but not always the term
		A lack of knowledge regarding care and treatment options
	Cognitive and interpersonal skills	The overlap between core therapeutic skills and skills needed for SDM
		Negotiation and containment as ‘new’ skills needed for SDM
	Memory, attention, and decision making processes	The availability of options may affect what is presented to the young person and family
	Behavioural regulation	A lack of clarity around whether there are guidelines and protocols for SDM
		Reviews of treatment and goals, whilst considered important, are conducted sporadically
Opportunity	Environmental context and resources	Facilities not conducive to SDM
		Limited or a lack of psychological interventions for SDM
		Administration and time constraints that inhibit SDM
		Procedural influences stop SDM
	Social influences	Team members positively and negatively influencing decisions
		Dominating parents
Motivation	Professional role and identity	Shared decision making is something CAMHS clinicians ‘do’
		Overruling a young person’s wishes due to professional standards
	Beliefs about consequences	Shared decision making empowers young people and families
		Shared decision making takes too much time
		Shared decision making can make psychological problems worse
	Beliefs about capabilities	Feeling confident engaging in SDM
		Feeling less confident due to a lack of knowledge around options
	Emotion	Feeling overwhelmed which inhibits SDM

### Capability

#### Knowledge

##### An awareness of the philosophy of SDM but not always the term

The majority of clinicians in this sample were aware of SDM as a concept, and as such what SDM entailed. When asked to provide a definition, they outlined examples such as partnership, eliciting patient values and preferences, as well as identifying treatment options.

It’s about us not being the expert all the time, it’s about being very explicit about what we can offer, what we’ve got… asking them what helps, what’s been helpful, as well as what we automatically think we may be able to offer them…and not us always sitting in that medical expert model…to keep the parents and families as the experts in their own lives really (Mental Health Nurse 4).

However, some clinicians highlighted they would not use the term SDM. Despite not knowing or using the specific term, all the clinicians believed that the concept of SDM was something that they clinically practiced.

It’s been only recently that I have been told about this shared decision-making, the term specifically…. I know what the meaning is. I think we have used the shared decision issues [for] a very long time… I mean, when we see patients in our clinic, when we think about treatments, when we offer treatments … asking their opinions and taking that into consideration (Psychiatrist 2).

When asked what terms they use instead, some clinicians mentioned ‘*informed consent*’, (Psychiatrist 2). This concept, which is related to SDM, has some underlying similarities though does not aim to actively involve the patient, nor elicit values or preferences.

##### A lack of knowledge regarding care and treatment options

A particular barrier for the clinicians was a lack of knowledge regarding the available care and treatment options for patients. This was particularly prominent when it came to the resources available outside of CAMHS.

Five years ago, there were a lot more resources out there, you felt comfortable signposting outside, you had better links, and I think over the years, as things have dwindled, I find myself struggling to see what’s out there as well (Clinical Psychologist 5).

The clinicians reported that funding cuts to community resources and voluntary organisations meant they did not know where to signpost with regard to other services. Even when resources were available, the clinicians tended to be cautious in making referrals due to long waiting lists or not knowing the quality of the service.

The interviews with the clinicians also demonstrated that some were not aware of certain options within their own service.

I am just thinking of us trainees for example, moving from placement to placement, how are you aware of all the options available? Or if you are aware of the three options rather than the five, you’re only going to present the three that you know about (Trainee Psychologist 2).

This was true for trainees who were on rotation as part of their training, but it was also the case for new members of staff who had just joined the service. In both situations, the clinicians acknowledged the possibility of not knowing all of the options within their service.

#### Skills

##### The overlap between core therapeutic skills and the skills needed for SDM

When asked to outline the skills that were needed for SDM, clinicians suggested being ‘*open*’, (CBT Therapist 1) ‘*honest*’, (Clinical Psychologist 3) ‘*transparent*’ (Clinical Psychologist 2), and ‘*listening to young people and parents*’ (Psychiatrist 1) as being important.

I think the basics, just to be listening well, to be a little bit more involved—to be actually listening properly and using what is being said rather than make your own points around it. I think it’s just the ability to connect with the people they work with really; I think that’s the main thing. It’s just about rapport-building and being quite transparent in what you’re doing (Therapist 1).

The clinicians commented on how this overlapped with skills acquired as part of their professional training. Clinical Psychologist 2 outlined how training had helped to improve their SDM skills through ‘*thinking about how it looks and how it works*’ and adapting their skillset to work with different groups.

##### Negotiation and containment as ‘new’ skills needed for SDM

The clinicians discussed how SDM in child and youth mental health incorporates multiple stakeholders’ preferences, values and views, which could lead to disagreements between parties on how to proceed with care and treatment. To navigate this situation, clinicians identified negotiation and containment as key skills.

Containment and negotiation…the dance of reciprocity, two steps forward and four steps back, and onto the side, a little jiggy then move forward again…you need to be careful not to alienate one individual (Mental Health Nurse 4).

The clinicians worried that the loss of engagement with an individual due to a lack of inclusion was potentially very detrimental. They explained that a disengaged young person who had not been included in choosing their treatment may be less likely to participate in therapy. Conversely, by not including a parent, the clinicians worried that the parent may choose not to bring the young person back to a subsequent session.

#### Memory, attention and decision-making processes

##### The availability of options may affect what is presented to the young person and family

The clinicians acknowledged that as part of their role, they were responsible for outlining the different treatment options to young people and their families. However, the clinicians were divided about which options to make patients aware of. Some clinicians highlighted that they would only suggest options that were available within their service.

Something [treatment option] that we didn’t offer? I don’t think I would necessarily point that out (Clinical Psychologist 4).

The clinicians justified limiting the options to what was available by explaining that they did not want to rupture the therapeutic relationship, or make therapy more challenging, as then individuals may not be able to have their first choice of treatment.

Conversely, other clinicians spoke of making a conscious choice to inform individuals about all their options, regardless of whether they were available in the service or not.

I think information’s key, I think the whole thing that…families need to be given the information and they can make the decisions for themselves about where they go and what supports they might get, so I think they should [have all options] (Mental Health Nurse 2).

This, clinicians stated, was the ‘spirit’ of SDM, as young people and families had a right to choose to seek treatment elsewhere if they desired, be that within CAMHS or elsewhere.

#### Behavioural regulation

##### A lack of clarity around whether there are guidelines and protocols for SDM

There was confusion amongst the clinicians as to whether there were guidelines and protocols for SDM in child and youth mental health. No clinicians described reading explicit guidelines pertaining to SDM. However, some clinicians speculated that such documents could exist.

Well I don’t know [if there are protocols/guidelines], I would assume there might be (Mental Health Nurse 1).

Other clinicians did not believe there were any protocols or guidelines, but they cited similar documentation which may be useful to help embed or facilitate SDM, such as ‘*protocols around consent and all that sort of stuff*’ (Clinical Psychologist 1).

Some clinicians posited that having protocols or guidelines would be helpful particularly for more junior members of staff and others not familiar with SDM: ‘*it would be good to have something standard so that everybody can do it*’ (Psychiatrist 1). However, others, such as Clinical Psychologist 2, worried that clinicians would view a protocol with contempt, as they ‘*might then see it as something else they’ve got to do*’.

##### Reviews of treatment and goals, whilst considered important, are conducted sporadically

Consistent with models of SDM, the clinicians stated the importance of reviewing treatment progress and goals with young people and families to understand how the young person is progressing.

Reviewing progress is really important, that you review what is happening with that young person, because if you don’t review, you don’t know what has helped, how much progress has been made, and where you next need to go, your next step, so reviewing progress is very important, and making changes if needed (Mental Health Nurse 1).

Despite the perceived importance of reviews by clinicians, it was acknowledged that often these were not completed on time or only on an ad hoc basis.

I will ask about the next one, and the review as such, is usually every 6 sessions, sometimes that happens, sometimes that doesn’t, and sometimes its partial follow up, so it’s not very concrete at the moment (Psychiatrist 1).

### Opportunity

#### Environmental context and resources

##### Facilities not conducive to SDM

Many of the clinicians spoke of the facilities in CAMHS as not being conducive to SDM with young people and families. For some, this extended to the building in which their CAMHS was located. They explained that the appearance and ambiance of the building, which felt like entering an adult medical environment, contradicted the philosophy of young people being treated as equals when it came to having an appointment.

Our offices are grim. Especially for young people, it’s just horrible. I think because it looks office-y and it looks clinical and it looks really official, that wouldn’t exactly give you—it’s like going into your doctor’s office and being told that you’re an equal partner really; it doesn’t feel particularly believable (Therapist 1).

Several clinicians stated that their appointment rooms were not conducive to SDM with young people. Aspects related to layout, a lack of space or the temperature limited the amount of time clinicians wanted to spend there, resulting in shorter appointments in which different options may not be fully explored.

…You instinctively want to cut down on conversation to get out of there, out of the room, so its basic things like that (Clinical Psychologist 3).

##### Limited or a lack of psychological interventions for SDM

The clinicians frequently outlined that only certain types of psychological support were available within their services. Not offering particular treatments resulted in the clinicians feeling that families were ‘*getting a raw deal with what we can provide*’ (Therapist 1). In the most extreme cases, the clinicians felt that there was no decision to be made or shared, as the only choice was between the treatment being offered or no treatment at all.

For example, the family therapist within the community, there’s been a move to the eating disorder team…so for self-harm it is CBT or nothing (Clinical Psychologist 4).

On the other hand, even when different types of psychological support were available, access to them could be precluded by being placed on a long waiting list. This was often more pronounced when therapies were longer, specialist or more intensive.

We’re quite often having to have the conversation even if we do have something. Sometimes there’s a wait, which can be difficult for families if they’re wanting something, for example like play therapy, because it’s quite an intensive therapy, there’s always a waiting list (Clinical Psychologist 1).

This was seen by the clinicians as having an effect on SDM, as it resulted in families (who were often desperate and had already been on a waiting list) choosing the option with the shortest waiting time rather than the option that would fit best with their specific values or preferences.

##### Administration and time constraints that inhibit SDM

A lack of staff members and increased patient demand were perceived factors affecting SDM according to a majority of the clinicians interviewed. During the assessment process, clinicians spoke about having to get through everything needed in the session, leaving little time for SDM.

If you have a time constraint around assessment and you’ve got five bits of paper to fill out, you’re going to try your best to get the information from the client; you’re not necessarily going to be thinking about the client in the longer term, if that makes sense…so it might be that rash decisions get made or it’s a short-term decision rather than thinking about the child’s needs as a whole and how they would engage in that longer term (Clinical Psychologist 1).

Time constraints were not just limited to assessment appointments. A few clinicians also highlighted the shift from shared to more directive decision-making when clinics were busy to try to keep to time.

I try to do [SDM] without overrunning, but it becomes difficult to manage the rest of the cases, because one has taken over more than an hour, then you begin to feel your own anxiety gets in the way…you become more dominant in a way, to say this is what you should do, this is what will help you, and you become more directive I suppose (Psychiatrist 1).

##### Procedural influences stopping decision-making

Clinicians highlighted that a young person’s presenting problem could lead them down a specific treatment path, leaving few options to be explored when it came to SDM. For one clinician, this was particularly relevant in the ‘best practice’ pathway for anxiety and depression where CBT was the emphasised treatment modality in their service.

So for anxiety and depression… we’ve had quite a few people going for training—so, the anxiety and depression pathway. So within that is CBT, because we know that’s what evidence—because that’s what we’ve been trained in—… the evidence shows CBT is effective… now we’d say CBT [to young people and families for treatment] (Clinical Psychologist 2).

#### Social influences

##### Team members positively and negatively influencing decisions

All the clinicians in this sample felt that SDM was part of the service culture as well as part of their professional role. Clinicians highlighted speaking to colleagues to come up with ideas for options for treatment.

As a team, we often feedback cases to the team, or talk about a client that you know is struggling, or we’d like to get more ideas, can we talk about it as a whole team, then we’ll come back as a team and present that and talk about that and get a lot more ideas coming through about what we can do (Mental Health Nurse 2).

The majority of clinicians found this helpful. However, some of the clinicians questioned whether too much input from other professionals might be unhelpful, as a larger number of voices increased the probability that a young person could then be forgotten about.

So yes, certainly dynamics, relationship….I suppose, between other professionals and that can be within the team or outside the team as well. It’s sometimes hard to hold the young person in mind if you’ve got too many voices kind of going over them, really (Clinical Psychologist 2).

##### Dominating parents

Several clinicians reported that sometimes parents demanded their children be seen in CAMHS even if the assessment suggested they did not meet the threshold.

She’s pretty much dragged him to an appointment. So the care plan with my patient, I’m saying “What do you want to do?” and [the young person’s] like, “This is actually quite manageable really. I don’t really want to do anything about it,” whereas Mum’s really clear that it’s causing tension in the home and it can’t carry on; that she wants zero habits all the time (Therapist 1).

In such circumstances, the clinicians reported feeling obligated to keep these cases open, even though they felt that there was little they could do for the young person and family. This meant overriding the young person’s feelings or wishes to placate the parent.

### Motivation

#### Professional role and identity

##### Shared decision making is something CAMHS clinicians already ‘do’

All the clinicians interviewed mentioned they felt that SDM was a routine part of their practice that was intertwined with their role in child and youth mental health.

That’s always been my approach. And, to be honest, I try to do that with everything else, so for example, if I’m using CBT, I try to do so in a collaborative way, asking for their views and opinions (Trainee Psychologist 3).

The clinicians outlined that to develop an effective treatment and care package for young people and families, it was important to take into account their preferences and cultural values, as these were ‘*aspects in themselves, their culture and their lives*’ (Nurse 2).

##### Overruling a young person’s wishes due to professional standards

Whilst the clinicians acknowledged that SDM was part of their practice, many spoke of the professional boundaries and standards that needed to be upheld which were sometimes at odds with SDM. The clinicians frequently cited capacity issues around mental health difficulties which meant that a young person could make risky decisions which would not be in their best interests if they compromised their safety.

Someone’s decision making may be impaired due to a psychotic episode, low BMI [Body Mass Index] due to an eating disorder, or suicidal… and the decision of the child or young person may that they don’t want to go into hospital, or talk to the professional, but again it is about keeping the young person safe (Trainee Psychologist 2).

#### Beliefs about consequences

##### Shared decision making empowers young people and families

The majority of clinicians outlined a benefit of SDM with young people and families related to patient empowerment.

They are empowered, it helps therapy so much and in so many ways, that it moves the process that they are engaged in things that you are doing and the throughput happens (Clinical Psychologist 5).

Giving the patient a sense of autonomy and control to help meaningfully shape treatment was seen by the clinicians as crucial if care and treatment were ultimately going to be successful.

##### Shared decision making takes too much time

Time was an important factor for the clinicians when it came to SDM, with many feeling that it took longer to implement treatments involving SDM as opposed to a more paternalistic approach in which the clinicians made the decisions.

[SDM] could slow down the pace or slow down the work because you’ve got to work at their pace, when they’re ready to access and take in that information (Mental Health Nurse 3).

The clinicians felt that it could take longer to make decisions in certain circumstances or with particular individuals. This included situations with very young people, individuals with learning disabilities, when patients did not come prepared to make a decision or when there were multiple parties involved. However, not all the clinicians mentioned time as a factor that affects SDM, and one clinician felt that it actually did not take any extra time as it was ‘*not something that was separate*’ and should be ‘*built into normal practice*’ (Clinical Psychologist 1). Another clinician outlined that whilst SDM may initially take longer, it could result in more engaged and motivated patients over the long term which would then positively impact on the initial time invested.

Initially it is more time consuming, I don’t think that would bear out over time as it helps with motivation, but initially I think yeah (Clinical Psychologist 3).

##### Shared decision making can make psychological problems worse

One clinician outlined that SDM may not be in the ‘interests’ of the young person and family as they were likely to choose treatments which may minimise psychological distress or discomfort. The example here was specifically in relation to phobias, where the clinician described how not engaging in exposure therapy could exacerbate problems in the longer term.

When someone is behaving in a way which in the short-term might alleviate their anxiety, so, for example, ‘I don’t want to go outside because it’s scary’. Okay, in the short term that’s going to make the anxiety go away but then the behaviour reinforces and they stay in the house for six months. So the disadvantage of being person-centred and going along with their decision there is actually that sometimes they can remain stuck if they’re unwilling to engage in a therapeutic technique for which there is pretty good evidence works really well (Trainee Psychologist 3).

In the longer term, the clinician felt this could affect the young person’s chances of making a good recovery.

#### Beliefs about capabilities

##### Feeling confident in engaging in SDM

In most instances, the clinicians stated that they were capable and confident in engaging in SDM with young people and families.

…I feel comfortable and confident [in engaging in SDM], yes… (Psychiatrist 1).

This was often attributed to the concept being part of their professional training, as well as a culture within CAMHS which aims to foster SDM with young people and families.

##### Feeling less confident due to a lack of knowledge around options

A few clinicians outlined that they did not feel confident discussing particular psychological therapies or medications with young people and families.

There’s something in our service called cognitive analytic therapy and when I explain it to families I don’t really feel that confident because I’ve never really worked within that model or understand it that much. But I guess, that’s just about my learning that I need to go and speak with somebody and get more information and get people to help me understand it a bit more (Clinical Psychologist 4).

The clinicians conceded that to address this they needed to obtain more information so that they felt more comfortable talking about different therapies.

#### Emotions

##### Feeling overwhelmed which inhibits SDM

Many of the clinicians reported needing to be ‘in the right state of mind’ to engage properly in SDM with young people and families. The majority of clinicians in this sample reported feeling stressed and overwhelmed with the amount of work that they had to do and the number of patients they had to see. In some instances, this impacted on SDM, as clinicians could forget to mention the availability of some options.

Everyone’s really, really overstretched, overwhelmed. So maybe for some staff, what might happen is that they maybe think actually there could be this other treatment option that we could consider with the family, but then they might forget (Clinical Psychologist 4).

Other clinicians outlined how stress impacted on their ability to participate in SDM, as it stopped them from asking questions and listening to the young person and family, and instead they ‘prescribed’ the normal treatment without taking into account the values and preferences of the young person and family.

## Discussion

The aim of this study was to investigate healthcare professionals’ perspectives on the factors that affect SDM with young people who have internalising difficulties in the context of child and adolescent mental health services in the UK. The study drew upon the Theoretical Domains Framework, which has been widely used in other settings, but has not been used previously around SDM. Ten of the fourteen domains in the TDF [10] were identified in the interviews.

The skill of listening was identified by the clinicians as important in facilitating SDM, along with other skills such as honesty, openness, transparency and empathy. These findings are similar to the skills mentioned in previous research, in which listening, along with the need to respect and validate the young person and parents [20, 22], was identified. The majority of skills cited here appear to revolve around having more honest discussions with young people and families. This is aligned with the philosophy of SDM, within which both the clinician and the young person and family contribute skills, knowledge and experience to form partnerships [1, 2, 29]. The clinicians described skills related to SDM that were acquired during their professional training and could be honed and refined through supervision. ‘New’ skills which clinicians believed to be important centred around resolution, specifically negotiation and containment. Whilst negotiation has been mentioned as important in some other models [e.g., 3], containment appears to be a novel finding. This could highlight the unique situation that exists when working with children and young people of having multiple stakeholders involved in the process of SDM [30], each with their own values and preferences. This corresponds with findings indicating that discordant views are frequent between parents, young people and clinicians [31, 32].

The importance of a young person’s capacity to be involved in decision making was also highlighted in this study, and it has been a prominent finding in previous research [19, 21, 23, 24]. Within this context, clinicians explained that to protect a young person with limited capacity, they might overrule their wishes or preferences. However, findings in this study exclusively relate to capacity in relation to mental illness, with professionals citing cases of low BMI and suicidality. This contrasts with some of the literature that also includes professionals citing age as a factor when determining whether young people can be involved [21, 24], and it corresponds with assertions by other academics, such as Alderson and colleagues, that age is not a barrier to involvement [33–36].

A lack of information sharing has been highlighted as a barrier to SDM [24]. The findings from this study may shed some light on some of the potential reasons why information may or may not be shared. One possible reason identified is that professionals may not know what treatment options are available outside of the service. For professionals new to the service as well as trainees, they may also be unaware of the options available within their own services. An alternative explanation arising from the findings in this study may be that some professionals may not feel comfortable or confident outlining different treatment options. This was particularly the case around medication, but also existed for some types of psychological interventions where some professionals have little knowledge.

Both this study and previous research [24] have highlighted resource issues as factors that affect SDM. Finite resources meant that treatment options were either not available or had lengthy waiting lists prior to access. A consistent finding across this study and previous studies suggests a lack of time as a barrier to SDM [24]. The clinicians stressed that increased patient demand resulted in patients and families often being seen back-to-back, which meant that clinicians had little or no time to explore their options in depth. In some circumstances, this also led to a more directive, rather than shared, approach to decisions concerning treatment. Whilst time barriers are a common concern for clinicians across clinical contexts with regards to SDM [37, 38], the likelihood of SDM significantly increasing session time has been refuted by some researchers [39].

The confines of service regulations also appear to be a barrier to SDM. For example, the allocation of cases to particular clinical pathways could limit the treatment options recommended for particular presenting difficulties. Similarly, services imposing minimum entry criteria meant that even if a young person or parent wanted help or support, they would not be entitled to it from services for which they did not meet the threshold. The impact of service regulations corresponds with identified barriers to SDM, where adherence to rigid protocols and operating procedures result in young people not having their needs met and not receiving tailored treatment [23].

The roles of other team members as a facilitator to SDM appear to be a novel finding. This may be through clinicians asking colleagues for advice regarding cases they are unsure about, or alternatively, colleagues being used as a source of support when the assessing clinician does not feel comfortable discussing particular treatment options. It has also been previously documented that other professionals can be a barrier to shared healthcare decisions. However, this was specifically in relation to disagreements between professionals over courses of treatment [24]. In this study, rather than disagreements between staff being a barrier to SDM, the clinicians highlighted that involving other professionals could lead to situations where the young person and parents’ views became lost due to the number of individuals involved.

Previous research has also highlighted that the sexual side effects of medication were a barrier to SDM with young people, as clinicians felt uncomfortable discussing such topics [24]. Side effects were also highlighted by a few clinicians in the present study; however, this was in the context of clinicians feeling they did not have enough knowledge about side effects to adequately discuss them with the patient. Those sexual side effects have not been mentioned here could be due to differences in the age ranges worked with, as services in this study worked with young people aged 0–18 years, as compared with 12–25 years in the aforementioned study [24]. Alternatively, this may also reflect differences in the samples related to those who could prescribe medication, with the present sample consisting of two medics (13%), compared with nine (41%) medics interviewed for the Simmons [24] study.

A strength of this study is that it examines a wide range of clinicians’ views regarding SDM across two outpatient services in the UK that work with young people and families. This adds breadth to the current literature on the topic, as it outlines some of the commonalities regarding the barriers and facilitators to SDM, as well as identifying nuanced individual views which have previously not been captured.

A further strength of this study is the use of the TDF [10]. Rather than asking individuals what they believed to be the barriers and facilitators of SDM, a systematic approach examining fourteen domains and underpinned by theory was employed. This may help illustrate the full range of barriers and facilitators around SDM, rather than just the ones that were immediately apparent to participants during the interview. Moreover, a semi-structured approach to the interviews was undertaken, which involved asking patients to elaborate on answers and allowed for deviation from the set TDF questions. This allowed for a richer narrative to be formed and provided further context to the barriers and facilitators around SDM.

In addition to the strengths of the study, the limitations should also be considered. Whilst teams of clinicians attended the presentations, relatively few clinicians decided to take part in the interviews or focus groups. As a result, this may represent a subsample of the clinicians’ views within each service and may not be representative of other clinicians who chose not to be interviewed. For example, the clinicians who chose to take part in the study may have had a particular interest in SDM or believed they were particularly good at it and wanted to share their experiences.

The use of the TDF [10] may also be considered a limitation. Whilst it covers fourteen domains of barriers and facilitators collapsed from the theory, it is possible there are others not covered by its current scope. Additionally, as the framework is fairly new, it may also be refined over time, with new domains being created under which the barriers and facilitators may fall. This can be evidenced by the two additional domains that have been added since its first conceptualisation [10].

## Conclusion

A number of barriers and facilitators have been identified as affecting SDM, both with young people who have internalising difficulties and their parents. Interestingly, many of these overlap with the findings from previous literature. These included skills such as listening, the young person having limited capacity due to mental illness, not knowing what options are available, and finite resources. Novel findings included: containment as a skill, clinician uncertainty over the term SDM, the use of team members to help offer suggestions when the clinician was stuck, and for one clinician a concern that SDM could make existing difficulties worse. This appears to be the first time these have been mentioned in relation to the wider literature on SDM in mental health.

Understanding what clinicians believed the barriers and facilitators to be, could help inform future interventions. For example, leaflets could be made for all new clinicians on what options are available within their services. However, other barriers, such as room and building layout, access to different treatments and more time to spend on SDM, are more challenging to implement, and so need to be taken into account when considering the experience of SDM in the ‘real world’.

## Electronic supplementary material

Below is the link to the electronic supplementary material.

Supplementary material 1 (DOCX 15 kb)

Supplementary material 2 (PDF 509 kb)
