# Supplementary material for: Barriers and facilitators to shared decision making in child and youth mental health: clinician perspectives using the Theoretical Domains Framework
Source: Eur Child Adolesc Psychiatry. 2018 Sep 18;28(5):655–66. doi: 10.1007/s00787-018-1230-0 (PMC6514112; doi:10.1007/s00787-018-1230-0)
Supplement: Supplementary file 1 — Supplementary material 1 (DOCX 15 kb) [file 787_2018_1230_MOESM1_ESM.docx]

Introductions

- Introduce self
- Talk about organisation and research
- Data protection
- Safeguarding information

Demographic information

- Can you tell me about why you went to CAMHS (diagnosis)?
- Can you tell me how long you have been in CAMHS (length)?
- Gender
- Age
- Ethnicity

General

- In your experience of attending CAMHS, how often have you been involved in decisions about your/your childs care/treatment?
- *Can you give me examples? (if have)*
- *How does this make you feel?*
- *What types of decisions would you like to be involved in?*
- *If I used the term shared decision making, what do you think it means?*

1. Knowledge

- From your experience, did you know that you/your child could be involved in decisions about your care and treatment?
- If so, how did you know about this, do you have examples?

1. Skills

- In your experience, what skills are needed by young people and parents to engage with healthcare professionals in decision making?
- In your experience, what skills are needed by clinicians to engage young people and parents/guardians in decision making?

1. Memory/attention/decision making processes

- In your experience, do you ever forget to ask questions or remember decisions about your/your childs care and treatment?
- In there anything that would help this?
- Do you find it easy or difficult to pay attention fully or hold in mind what has been discussed about your/your child’s care? Can you tell me more?

1. Behavioural regulation

- In your experience, how do you monitor if you have been involved in shared decision making? Can you tell me more?

1. Environmental context and resources

- What types of environments are conducive to shared decision making?
- Who needs to be there? Where? Time allocated?

1. Social Influences

- Do other people ever affect your decision making about your/your child’s care? In what ways?
- Have you experienced any conflict with health professionals over decisions about care?
- What do you think of the communication about care between clinicians and families/young people?
- How do you think clinicians feel about decision making with young people and parents/guardians?

1. Social Role

- Whose responsibility is it to make decisions about a young person’s care?
- In your experiences, do you think that certain healthcare professionals are better suited to shared decision making, can you tell me more?

1. Beliefs about capabilities

- Do you feel that you are able to be involved in decisions about your/your childs care? Can you tell me more?
- What would help you feel more comfortable/confident in making decisions about your care?
- What would make it easier to make decisions in your/your childs care?

1. Optimism

- How confident/optimistic are you that you are able to be initiate/implement shared decision making?
- How confident/optimistic are you that services are able to be initiate/implement shared decision making?

1. Beliefs about consequences

- What do you think are the benefits/disadvantages of young people and families engaging in decisions about their care, do you have examples of from the care you received, can you tell me more?
- What do you think happens when young people and families are more involved/not involved in their care?

1. Intentions

- Do you/your child intend to be involved in decision making? Can you tell me more?

1. Reinforcement

What incentives are there for you/your child to engage in shared decision making? Personal/financial?

1. Motivation and Goals

- From your experience, why do you think yourself/young people would like more of a say in their care?
- From your experience, are there any particular decisions that you feel are more important for young people and parents/guardians to be involved in?
- From your experience, are there any particular decisions that you feel are less important for young people and parents/guardians to be involved in?

1. Emotions

- In your experience, do you think yours or your childs emotions affect decision made about your care? In what ways? What kind of emotions?

**Thank you.**

- Outline how data will be processed,
- What will be done with the data
- How they can contact the researcher for more information
